# Secondary Metabolites Composition and Their Histochemical Localization in the Fruit of *Piper malgassicum* Papini, Palchetti, Gori and Rota Nodari (Piperaceae)

**DOI:** 10.1002/cbdv.202403289

**Published:** 2025-07-05

**Authors:** Sara Falsini, Sara Ballantini, Alexander Pittella, Gelsomina Fico, Claudia Giuliani, Enrico Palchetti, Massimo Gori, Stefano Biricolti, Emilio Corti, Alessio Papini, Luca Calamai, Marzia Innocenti

**Affiliations:** ^1^ Dipartimento di Biologia Università degli studi di Firenze Florence Italy; ^2^ Dipartimento NEUROFARBA Sezione di Scienze Farmaceutiche e Nutraceutiche Florence Italy; ^3^ Dipartimento di Scienze Farmaceutiche ‐ DiSFARM Università di Milano Milano Italy; ^4^ Orto Botanico G.E. Ghirardi – DiSFARM Università degli Studi di Milano Toscolano Maderno Italy; ^5^ Dipartimento di Scienze e Tecnologie Agrarie Alimentari Ambientali e Forestali (DAGRI) Università degli Studi di Firenze Florence Italy; ^6^ Centro Interdipartimentale di Servizi per le Biotecnologie di Interesse Agrario Chimico e Industriale (CIBIACI)‐Università degli Studi di Firenze Florence Italy

**Keywords:** alkaloids, idioblast, linalool, micromorphology, *Piper malgassicum*, piperine, terpenes, α‐phellandrene

## Abstract

The anatomy, histochemistry, and secondary metabolite composition were investigated on the drupes of *Piper malgassicum*, one of the components of the spice voatsiperifery pepper. The high amount of piperine recorded with high‐performance liquid chromatography with photodiode‐array detection analysis was localized with histochemistry mainly along the fruit mesocarp in idioblasts, since they were positive to alkaloids (Wagner reaction). The fruit was analyzed at different stages of maturation. β‐carotene reached the highest concentration when the drupe was at an intermediate stage of maturity (orange color) and maintained the same concentration also at full maturation (red color). The terpenes fraction present in higher amounts decreased with fruit ripening (as also piperine). Terpenes are presumably at their maximal concentration at the middle stage of fruit ripening to avoid damage to the pericarp by fungi and bacteria, while piperine would avoid feeding by animals. The reduction in concentration of these two components is linked to the seed dispersal stage after full fruit maturation. Both the most abundant terpenes, α‐phellandrene and linalool, show antimicrobial properties. These compounds are also known for anti‐inflammatory properties in vivo and in vitro, and hence, this plant may have medicinal properties. Since the total amount of terpenes is highest at an intermediate stage of fruit maturation, the optimal timing of collection would be prior to full maturation.

## Graphical abstract

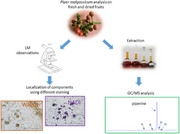

## 1 Introduction

*Piper* L. is a large genus including more than 2000 tropical species [1, 2]. This genus belongs to the “paleoherbs”, a group of families traditionally considered in an intermediate phylogenetic position between Monocots and Dicots [3].

This phylogenetic position is reflected in some peculiar vegetative arrangements that are somehow unusual both for Monocots and Dicots, as, for instance, the presence of a double circle of vascular bundles in the primary structure of the stem [4, 5]. The highest number of species has been described in America, followed by Southeast Asia and Australia [6, 7]. On the contrary, Africa is the continent where fewer species of *Piper* have been described [5], with only two native species for continental Africa, *P. guineense* Thonn. and *P. capense* L.f.The former is more closely phylogenetically related to *Piper nigrum* L. The real number of species present in Madagascar (and in the Mascarene Islands) is surely higher than with respect of continental Africa. However, the exact number is not easy to assess, since some species have been described many years ago and on the basis of a single herbarium sample [4]. A partial list comprises: *P. heimii* C. DC, *P. pachyphyllum* Baker, the recently described *P. malgassicum* Papini, Palchetti, Gori, Rota Nodari, and *P. tsarasotrae* Papini, Palchetti, Gori, Rota Nodari, while *P. borbonense* (Miq.) C. DC., described for the island La Reunion, at the time Île Bourbon [8, 9]. *P. malgassicum* and *P. tsarasotrae* were found to be phylogenetically related to *P. nigrum*. In addition, they are also traditionally used in Madagascar to produce spice (probably together with *P. borbonense*): the voatsiperifery pepper [4]. *P. malgassicum* is a humid forest species of Madagascar, probably more widely distributed than officially indicated. It is a dioecious liana climbing up to 10–15 m, and the fruit collection is done by hand [4]. A long list of medicinal and nutraceutical properties has been attributed to *P. nigrum*, particularly about menstrual and ear‐nose‐throat disorders in humans and for gastrointestinal diseases in livestock [10]. Therefore, it is important to investigate the terpene profile of *P. malgassicum* to make a comparison with that of *P. nigrum*, thus drawing indications regarding its potential therapeutic use.

Secondary metabolites on the vegetative parts of *P. malgassicum* were investigated and localized by histochemical analysis in leaves [11]. Alkaloids were specifically found in the mesophyll, within idioblasts, and terpenoids were localized both in the mesophyll and the glandular trichomes [11], while the fruit had not yet been investigated. The fruit is the most relevant part used as food in the family Piperaceae in general, and, despite having been often described as a berry, it is a drupe [12]. Since the fruit is the part of the plant used as a spice, a knowledge of its phytochemical features could be of particular interest to assess the main components of the *Piper malgassicum* and hence of the voatsiperifery pepper. For this reason, the aim of this study was to estimate the secondary metabolite content of the drupe and to localize its position with histochemical methods. Moreover, the drupes were collected at different stages of maturation, and the phytochemical analysis was performed on the volatile and non‐volatile fractions to estimate if the secondary metabolites change their composition during the process.

## 2 Results

### 2.1 Micromorphological Analysis

**FIGURE 1:**
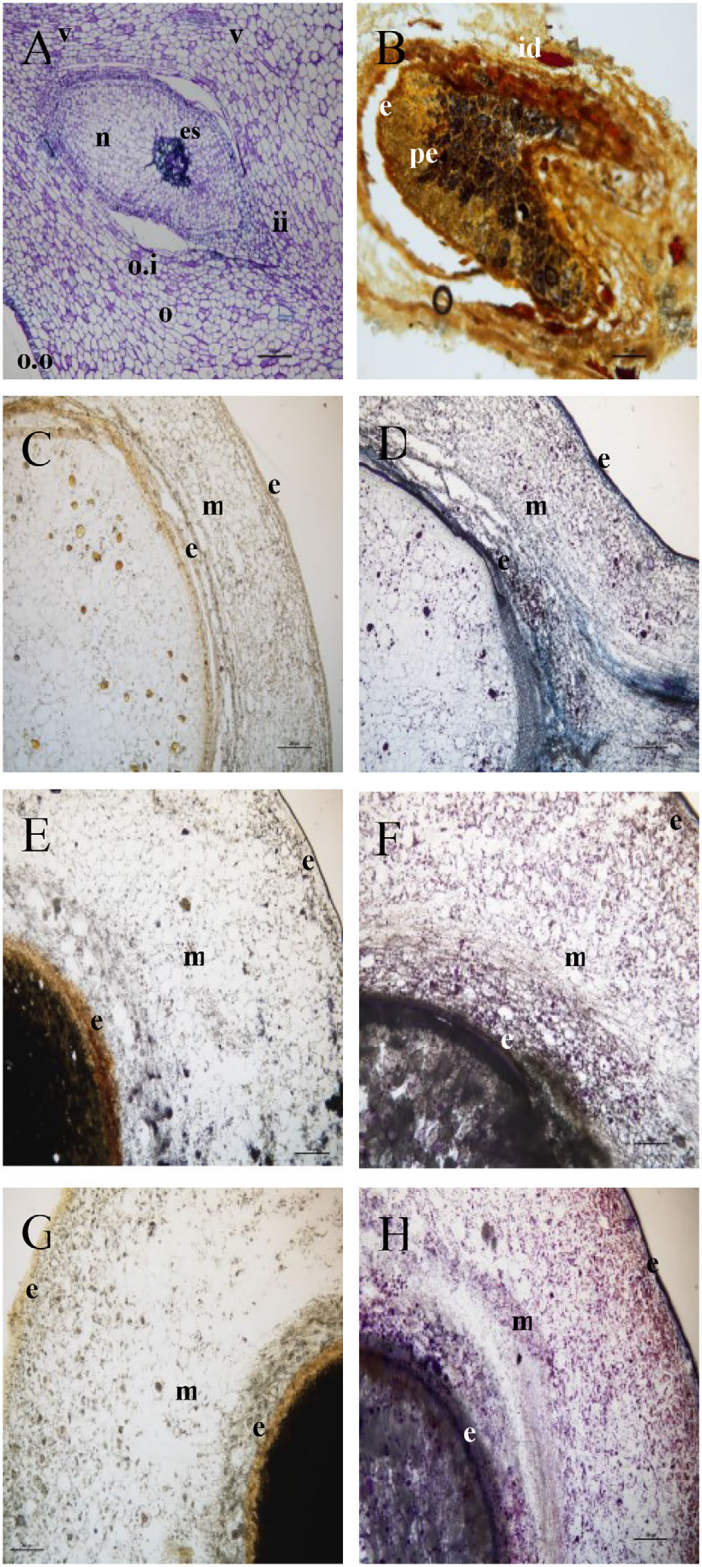
(A–G) *Piper malgassicum* ovary/drupe cross‐section. (A) Toluidine blue staining. Drupe at the later stage of maturation. Bar = 100 µm. (B) Wagner staining on a dried mature drupe. Bar = 100 µm. (C) Wagner staining on unripe fruit. Bar = 200 µm. (D) NADI staining on unripe fruit. Bar = 200 µm. (E) Wagner staining on ripe fruit. Bar = 200 µm. (F) NADI staining on ripe fruit. Bar = 200 µm. (G) Wagner staining on overripe fruit. Bar = 200 µm. (H) NADI staining on overripe fruit. Bar = 200 µm. ep = endocarp; ep = epicarp; es = embyo sac; ex = exocarp; idb = idioblast; ii = testa; m = mesocarp; nu = nucellus; o.it = internal ovary integument; o.ot = outer ovary integument; op = ovary parenchyma; ov = ovule; per = perisperm; vb = vascular bundle. Two replicates for each sample were examined.

The anatomy of mature fruit is shown in Figure 1A, where the ovule is developing into a seed, and a gap between the internal and the external ovule integument is forming (Figure 1A). The nucellus appears still vital, apart from the central part of the ovule (Figure 1A). The Wagner test for alkaloids applied on sections of the dried fruit showed that some cells of the exocarp and mesocarp parenchyma (idioblasts) were stained positively (Figure 1B). The alkaloids appeared to be particularly abundant also close to the ovule integument and in the part of the mesocarp closer to the ovule and underneath the pericarp. Cross‐sections obtained at the three stages (unripe, ripe, and overripe) of ripeness showed positive regions to Wagner staining (Figure 1C,E,G). In particular, at the immature stage, the alkaloids were found mainly in the mesocarp and the internal integument of the ovule. At the two latest stages of development, starch grains were abundantly detected inside the endocarp, while alkaloids were observed only in the mesocarp (Figure 1F,G). Furthermore, the mesocarp and the internal tegument were characterized by the presence of terpenes, evidenced by the NADI reagent at the different stages of fruit development (Figure 1D,F,H). Terpene content increased with the maturation process, especially in the endocarp of the fruit. The transversal section of the unripe fruit showed few droplets inside the endocarp.

**FIGURE 2:**
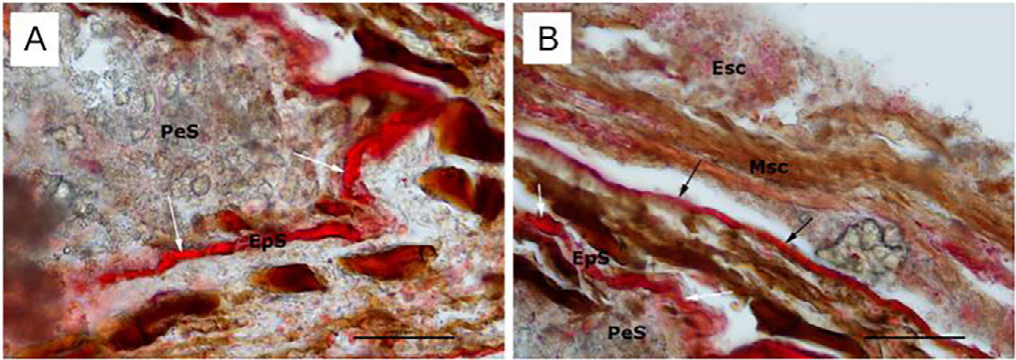
Ripe fruit cross‐section of *Piper malgassicum*. (A) A Sudan‐positive layer (Eps) surrounds the perisperm (white arrows). (B) Sudan‐positive aggregates are present in the exocarp and in the mesocarp (arrows). Bar = 50 µm. Esc = exocarp, Eps = episperm), Msc = mesocarp, PeS = perisperm. Two replicates for each sample were examined.

Lipid aggregates, slightly positive to Sudan III‐IV, were randomly distributed in the perisperm (Figure 2A) and the mesocarp (Figure 2B). In particular, a lipid layer is evidenced in the mesocarp region (black arrows in Figure 2B). Sudan III‐IV stained episperm resulted in a homogeneous hydrophobic stratum surrounding the perisperm (Figure 2B).

### 2.2 Phytochemical Analysis

#### 2.2.1 Non‐volatile Fraction

**FIGURE 3:**
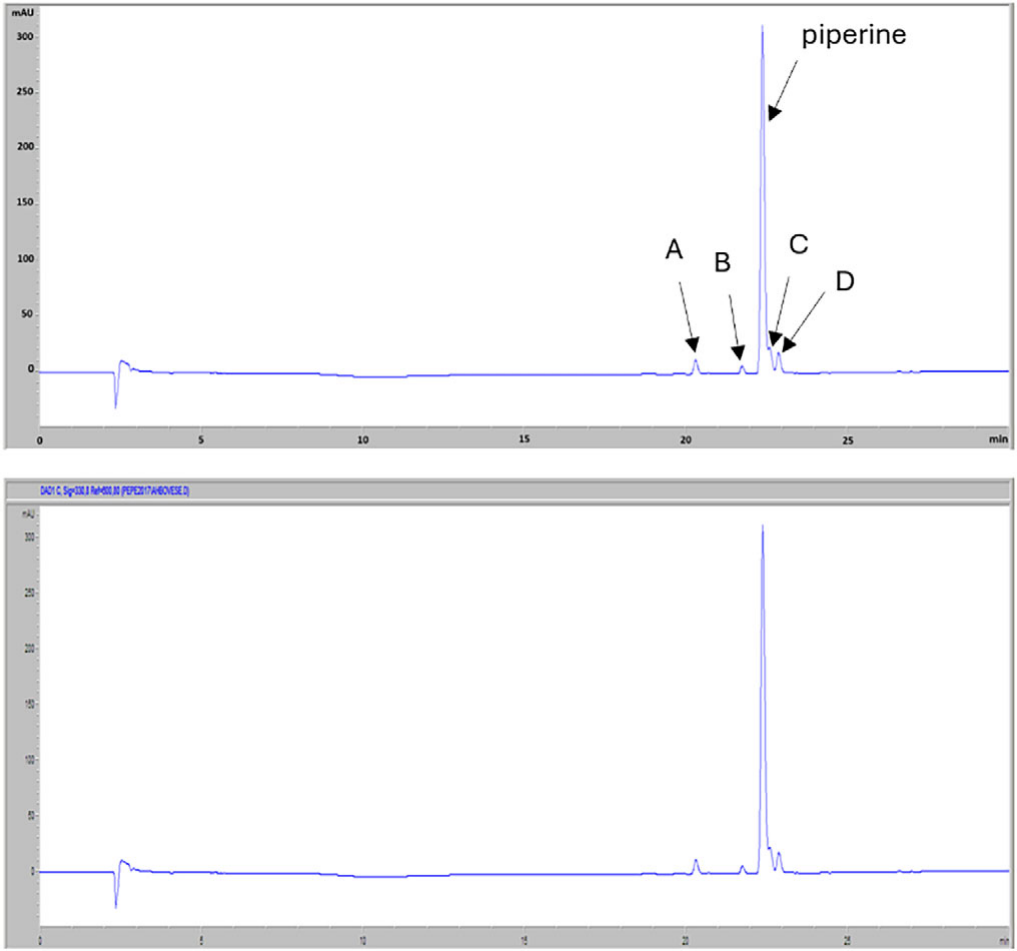
High‐performance liquid chromatography with photodiode‐array detection (HPLC‐DAD) profile of piperine (the highest absorption peak)with four piperine isomers (peaks A, B, C, and D at the following retention times 20.16, 21.6, 22.45, and 22.73 min) with absorption spectra close to the piperine.

The high‐performance liquid chromatography with photodiode‐array detection (HPLC‐DAD) profile at 330 nm (Figure 3) of the non‐volatile fraction from *P. malgassicum* drupe extracts showed piperine as the main compound, which was identified by comparison of retention time and the UV‐Vis spectrum with the commercial standard. In literature, in piper drupes, other alkaloids such as 4‐5 dihydropiperine, pipernonaline, piperlongumin and piperlonguminine, which have similar chromophoric groups, but very different molecular weights, were reported [13, 14]. Furthermore, piperine (*E*,*E*‐*trans‐trans*‐piperine) is known to easily and fairly quickly undergo photo‐isomerization, giving rise to the following three isomers: *Z*,*E‐*(*cis‐trans*)‐piperine (isopiperine); *E*,*Z*‐(*trans‐cis*)‐piperine (isochavicin); *Z*,*Z*‐(*cis‐cis*)‐piperina (chavicina) [15].

In analyzed samples, the piperine content ranged from 80% of the total alkaloids in the green drupe to 83% in the red drupe, and up to 85% in the orange drupe.

**FIGURE 4:**
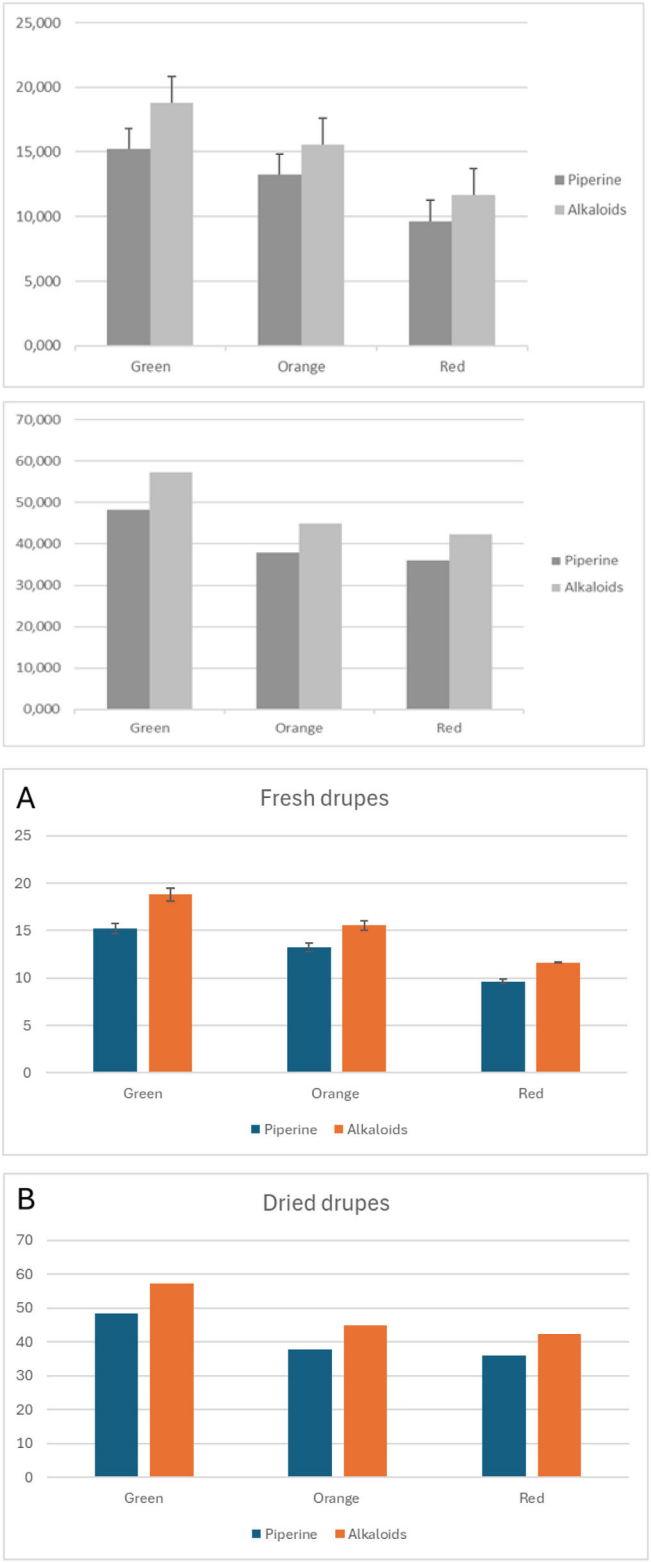
Piperine and total alkaloid content expressed as mg/g of fresh piper drupes (two replicates) (A) and dried piper drupes (one replicate) (B).

As reported in Figure 4A,B, the piperine content of *P. malgassicum* decreased with the ongoing maturation process. The amount of piperine was 4.7 mg/g in green fresh drupes and 9.43 mg/g in red fresh drupes. The piperine content was 48 mg/g in green dried drupes and 35 mg/g in red dried drupes. Although the data regarding the piperine amount analyzed in dried drupes are preliminary, we detected a decrease in the piperine amount equal to 36% and 27% in green and red drupes, respectively, following the drying process.

The amount of piperine and its derivatives in fresh drupes was, as expected, about three times lower than in dried drupes due to the water content. The ratio of piperine/total alkaloid content remained constant. However, it was possible to make a comparison between the dried samples and the freeze‐dried samples from fresh pepper to evaluate if the drying process had led to a possible degradation of the alkaloids. The quantitative data of the freeze‐dried samples were comparable to dried samples, so the adopted drying condition did not result in a loss of non‐volatile components.

#### 2.2.2 Volatile Fraction

Volatile fraction revealed the presence of two main classes of compounds: monoterpenes and sesquiterpenes. The relative percentages of monoterpenes and sesquiterpenes compounds found in the fresh drupes harvested at different stages of ripeness (green, orange, and red) in the Ambohimitombo locality are reported in Tables 1 and 2, respectively.

**TABLE 1:** Monoterpene components identified by gas chromatography/mass spectrometry (GC/MS). The percentage is relative to the integration of the peak area and is calculated with respect to the sum of all the peaks. The relative percentages are, hence, to be considered approximate, and the analysis is only semi‐quantitative.

Sample	RT^a^ (min)	Relative abundance (%)		
		Green	Orange	Red
α‐pinene	9.831	9.868	6.640	7.805
β‐pinene	12.247	23.473	11.540	11.057
sabinene	12.570	4.350	8.535	7.560
δ3‐Carene	13.393	4.573	5.083	2.422
myrcene	13.643	2.235	1.912	1.787
α‐phellandrene	13.876	29.528	43.062	43.765
β‐phellandrene	15.193	17.698	14.190	16.598
Limonene	14.860	5.454	5.706	5.601
β‐ocimene	16.213	2.231	1.140	1.356
p‐cymene	16.934	0.104	0.177	0.228
terpinolene	17.299	0.397	0.561	0.452
linalool	24.091	0.089	1.456	1.371

^a^ Retention time.

**TABLE 2:** Sesquiterpene components identified by gas chromatography/mass spectrometry (GC/MS). The percentage is relative to the integration of the peak area and is calculated with respect to the sum of all the peaks. The relative percentages are, hence, to be considered approximate, and the analysis is only semi‐quantitative.

Sample	RT (min)	Relative abundance (%)		
		Green	Orange	Red
α‐cubebene	22.388	0.284	0.235	0.457
α‐ylangene	23.225	3.775	1.246	2.265
α‐copaene	23.479	1.728	1.067	3.796
β‐ylangene	25.594	1.073	0.360	0.976
β‐Elemene	25.958	19.426	5.615	9.628
*cis*‐muurola 4,(14) 5 diene	26.177	3.884	3.457	2.199
β‐caryophyllene	26.311	50.196	18.704	40.830
γ‐elemene	27.069	2.865	0.129	0.991
germacrene D	27.302	0.382	0.106	0.281
β‐farnesene	27.434	1.412	0.351	0.942
aromadendrene	27.528	1.596	0.383	1.222
α‐caryophyllene	28.077	3.515	4.126	5.354
γ‐muurulene	28.334	0.906	1.667	2.156
α‐muurulene	28.847	0.156	0.033	0.254
β‐bisabolene	28.984	22.018	10.457	23.905
β‐selinene	29.245	1.972	0.241	7.995
α‐selinene	29.346	1.720	5.340	2.502
germacrene A	29.508	1.483	0.536	2.895
γ‐cadinene	29.903	0.370	1.049	1.795
germacrene B	31.732	0.134	0.043	0.124
4 epi cubedol	32.739	0.687	3.350	2.263
cubedol	33.863	1.281	11.230	8.234
ledol	35.677	0.509	1.163	0.795
germacren d4‐ol	35.863	7.918	11.261	10.789
elemol	36.177	3.145	20.699	9.520
γ eudesmolo	38.363	0.442	0.010	0.016
β‐eudesmol	38.537	1.258	3.633	4.603

RT = Retention time.

Regarding the monoterpenes, 12 compounds were identified in the fruits at the three stages of ripeness as shown in Table 1. In particular, α‐phellandrene dominated the profile (29.528%–43.765%), followed by β‐phellandrene (14.190%–17.698%), β‐pinene (11.540%–23.473%), sabinene (4.350%–8.535%), and α‐pinene (6.640%–9.868%).

**FIGURE 5:**
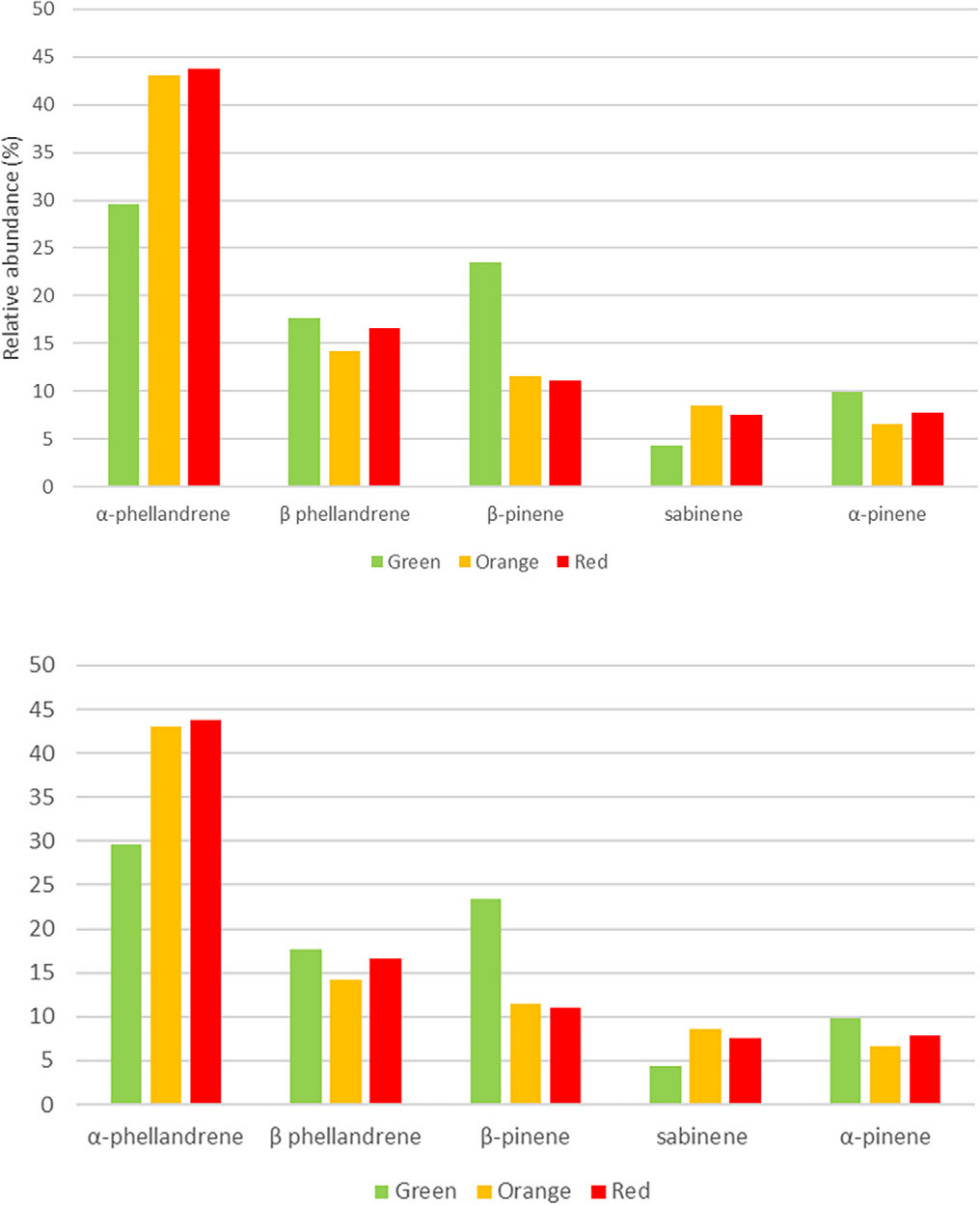
Relative abundance of the five most abundant monoterpenes in the fruits at the three stages of ripeness (green, orange, and red).

The relative abundance of the five most representative monoterpenes varies at the three stages of fruit ripeness. For example, in the green fruits, the profile was dominated by α‐phellandrene (29.528%), followed by β‐pinene (23.473%), β‐phellandrene (17.698%), α‐pinene (9.868%), and sabinene (4.350%). As shown in Figure 5, the relative percentages of α‐pinene and β‐phellandrene are almost constant in the three stages of ripeness. β‐pinene decreases in the orange and red stages up to 11%, while sabinene shows the highest content at the intermediate ripening stage with a relative percentage of 8.535. In particular, there is an increase in the production of sabinene (≈ 2 times) and α‐phellandrene (≈ 2 times) in the last two stages, reaching 8.535% and 43.765%, respectively.

Furthermore, the gas chromatography/mass spectrometry (GC/MS) profile of the *P. malgassicum* fruits revealed 27 sesquiterpene components, as shown in Table 2. The most abundant terpene was β‐caryophyllene (18.704%–50.196%), followed by β‐bisabolene (10.457%–22.018%), elemol (3.145%–20.669%), β‐elemene (5.615%–19.426%), germacren d4‐ol (7.918%–11.261%), and cubedol (1.281%–11.230%).

The relative abundance of the six most representative sesquiterpenes varies at the three stages of fruit ripeness. For example, in the green fruits, the profile was dominated by β‐caryophyllene (50.196%), followed by β‐bisabolene (22.018 %), β‐elemene (19.426%), germacren d4‐ol (7.918%), elemol (3.145 %), and cubedol (1.281%).

**FIGURE 6:**
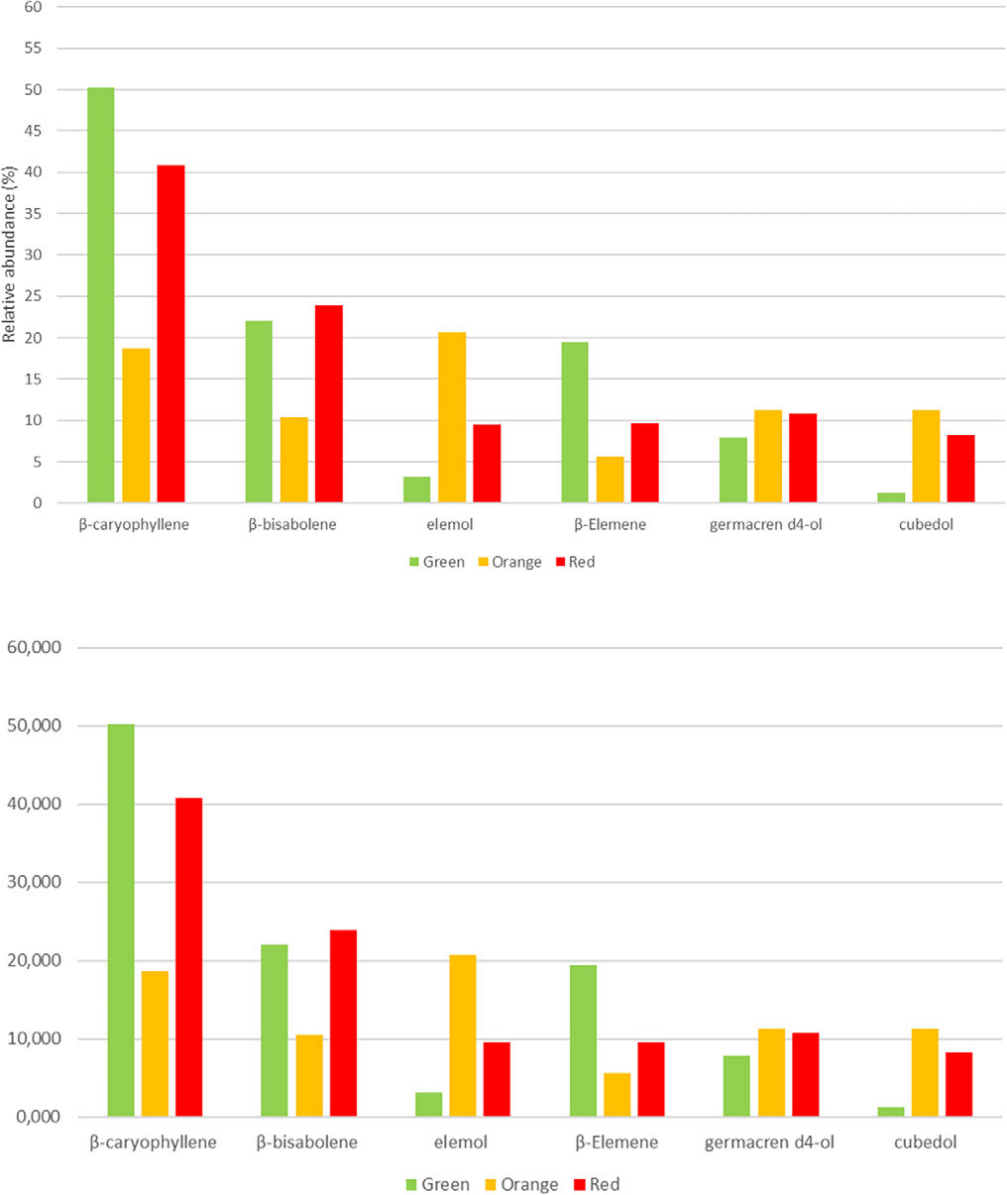
Relative abundance (%) of the six most abundant sesquiterpenes in the fruits at the three stages of ripeness (green, orange, and red).

As shown in Figure 6, the relative percentage of germacren d4‐ol is almost constant in the three stages of ripeness, ranging from 7.918% to 11.216%, while β‐elemene decreases in the orange and red stages up to 6%–9%. Elemol shows the highest content at the intermediate ripening stage with a relative percentage of 20.699. In particular, there is an increase in the production of cubedol (≈ 10 times) in the last two stages, orange and red, with 11.230% and 8.234%, respectively. β‐Caryophyllene and β‐bisabolene showed the highest values at the green and red stages.

**FIGURE 7:**
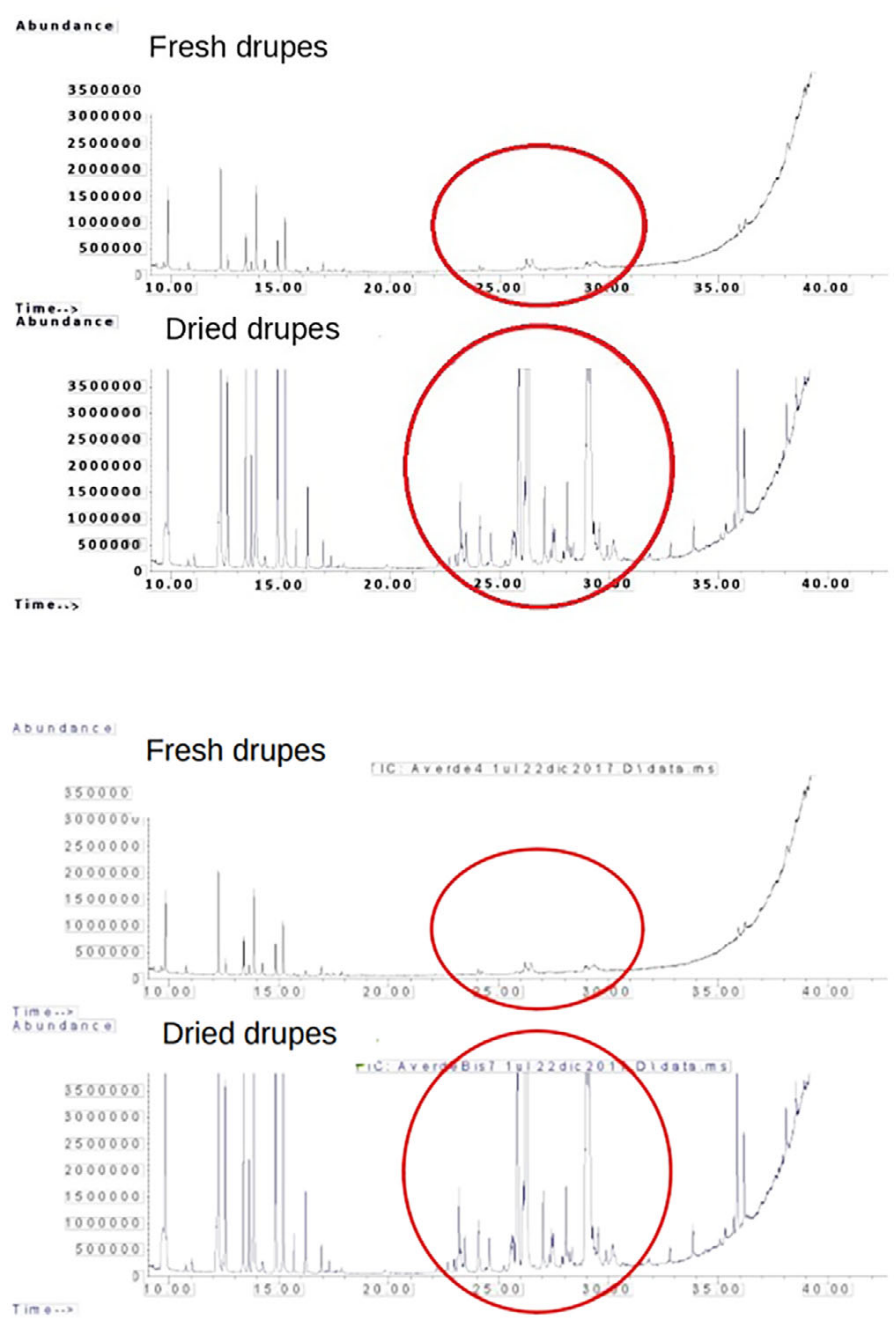
Results of high‐performance liquid chromatography with photodiode‐array detection (HPLC/DAD) analysis on terpenes. Above are the results of the analysis on the fresh drupe, and below are the results of the analysis on the dried drupe. Sesquiterpenes are more abundant in dried specimens.

The drying process resulted in an increase in sesquiterpenes versus monoterpenes, which probably evaporated in part during the heating, so in the fresh samples, the peaks related to sesquiterpenes are less intense in the dried samples, as shown in Figure 7.

## 3 Discussion

The drying process resulted in an increase in sesquiterpenes versus monoterpenes, which are more volatile molecules and were lost in part by volatilization during the heating process. Thus, in the fresh samples, the peaks related to sesquiterpenes are less intense compared to the dried drupes. In the last, in fact, sesquiterpenes increasing may probably be due to the formation of new byproducts from the volatility of monoterpenes [16].

The observation of the anatomy of the ripe ovary after fertilization showed that only a single ovule was present inside the ovary; moreover, the tissues of the ovary walls were quite similar to the final epicarp‐mesocarp‐endocarp pattern to be seen in the fruit. This organization is the same as in *P. nigrum* and of most species of the genus *Piper* that were investigated anatomically [17, 18]. The most abundant amount of nutrients was stored in the perisperm, as in most species of Piperaceae until now investigated [17, 18]. The normal degeneration of the nucellus by programmed cell death [19, 20] is here limited to the part of the ovule closer to the developing embryo, while the rest of the parenchyma acquires the function of nutrient storage (perisperm) for the developing seed [18]. In *Peperomia* and other Piperaceae, apparently no degeneration of the nucellus occurs, and starch is accumulated in the parenchyma starting close to the developing embryo [18]. Hence, the situation in *P. malgassicum* appears to be intermediate between the most primitive one in Angiosperms [21] (with a fully functional endosperm) and that in *Peperomia* and other investigated species of genus *Piper* (where the nutrients are stored in the perisperm derived from the nucellus).

The parenchyma of the mesocarp appeared to stain not homogeneously in all the cells, both with toluidine blue and with the Wagner staining for alkaloids. The reason is the positivity of some single cells (idioblasts) of the mesocarp in the Wagner reaction. Also, the epicarp and the ovule integument appear weakly Wagner positive. We can hence conclude that the alkaloids in the drupe of *Piper malgassicum* are stored inside idioblasts of the mesocarp and, in lower amounts, in the epicarp and in the external ovule tegument. Lee et al. [22], in general, already observed the localization of piperine mainly at the level of the pericarp in *P. nigrum*, playing a role in the effective defense against biotic stressors [23]. The lack of this alkaloid in one of the Costa Rican species, *Piper cordulatum*, is correlated with the lack of biological activity in the insect bioassay [24]. Since piperine is the most important secondary metabolite to which pungency and pharmacological properties are attributed [25, 26], we can guess that also in *P. malgassicum* this compound has the same function that it has in *P. nigrum*. Thus, the spice voatsiperifery pepper will have properties somewhat similar to those of *P. nigrum* from the point of view of pungency.

The progressive reduction in piperine amount observed during the progressive ripening of the drupe is probably due to the increase in the dimension of the fruit during the ripening process. Apparently, piperine is already present in the ovary, reaching its higher amount at the green stage. This alkaloid is a fundamental component of the defence system of the plant; it probably has the function to select the right animals for seed dispersal, diminishing the feeding on the drupe by other less efficient dispersers and avoiding feeding on the fruit too early in time [27, 28]. As a matter of fact, animals adapted for seed dispersal for species of genus *Piper* were inhibited in their action by piperine in the Neotropics [29]. At the moment, knowledge about animals relevant for seed dispersal (and the same for pollinators) in *P. malgassicum* is lacking. In the genus, apparently, the most important fruit dispersers are bats in the Neotropical species [30, 31], while a higher variability with a prevalence of birds may be possible in the African and Asian species, where colored fruits are more common [32]. The red color of the ripe fruit of *P. malgassicum* leads us to guess more of an avian identity as a seed disperser since normally bat‐dispersed fruit are not well colored due to the night habits of these mammals [33].

The presence of resin ducts observed in the mesocarp may explain the recorded mono and sesquiterpenes identified with HPLC/DAD. Since this type of anatomical structure always contains terpenes [34]^.^

Other authors observed the presence of oleoresin (as Oleoresin Black Pepper) in the fruit mesocarp of *P. nigrum* [35, 36], but without an exact localization of the oleoresin in specific anatomical structures. Our results show that the presence of resin ducts may explain the high level of terpenes content that is related to the taste and scent of the commercial black pepper and also to that of the voatsiperifery pepper, of which *P. malgassicum* is one of the main components.

The most important terpenes (quantitatively) in *P. nigrum* essential oils have been found to be 3‐carene, β‐pinene, sabinene, limonene, and terpinen‐4‐ol [37]. These terpenes are variable in quantity depending on the assayed population [38] and plant organs (seeds or leaves) [35]. In *P. malgassicum*, we found the most important terpenes already recorded in *P. nigrum*, but in a lower amount. The most abundant terpenes in *P. malgassicum* ripe drupes are α‐phellandrene, β‐phellandrene, and β‐pinene among monoterpenes and β‐caryophyllene among sesquiterpenes, all of them not prevalent in *P. nigrum*, while the 3‐carene amount was relatively low. The same results were also confirmed in other investigations about *P. nigrum* [39, 40, 41]. These differences in terpene relative abundances and composition are surely responsible for the differences in taste and scent of voatsiperifery in comparison to black pepper [11]. Particularly important is linalool, which is known to be a fresh, pungent, and flowery odorant [42]. While the most abundant terpene in *P. nigrum*, 3‐carene, is considered active against beetles [43], and the second most abundant one, β‐pinene, abundant also in *P. malgassicum*, has microbicidal activity against fungi and bacteria [44]. The most abundant monoterpene in *P. malgassicum*, α‐phellandrene, is mainly active against fungi [45], but might have antinociceptive properties in mammals [46]. Anti‐inflammatory, antibiotic, antioxidant, anticarcinogenic, and local anaesthetic activities [47] have been attributed to β‐caryophyllene, the most abundant sesquiterpene [48]. Among monoterpenes, α‐phellandrene and β‐phellandrene have antiinflammatory activity [49, 50] and an antifungal compound [51], as is β‐pinene [35].

Also, linalool is relatively high at the intermediate stage in *P. malgassicum*, decreasing with drupe maturation, and is hence related to the defense of the fruit when it is not ready for dispersal.

α‐Phellandrene is also known to attenuate inflammatory responses [40], as is also linalool [52], and hence *P. malgassicum* may own potential medicinal properties not present in *P. nigrum*.

The observed increase in β‐carotene passing from the early stage to the intermediate (orange) stage is related to the attraction of the seed disperser. The attraction activity is synchronized with fruit maturation, probably with the function to avoid too early dispersal, when the embryo is not yet ready.

## 4 Conclusions

*Piper malgassicum* exhibits variation in the concentration of piperine and terpenes corresponding to fruit maturation. The probable function is to avoid feeding on the developing fruit before the embryo is ready for seed dispersal. The presence of α‐phellandrene and β‐phellandrene may be considered a clue for possible anti‐inflammatory activities. Since these two substances (and piperine) decrease with fruit maturation, the collection time to maximize their concentration should be before full maturation.

Further research should be undertaken to deepen the characterization of the phytochemical profile, particularly regarding alkaloid content, to validate the pharmacological properties of the secondary metabolites and to clarify their ecological role in fruit dispersal. An investigation about the dispersal modes of *P. malgassiucum* in the field would improve the explanation about the biological/ecological meaning of the erpene mixture specific composition and of the decrease in piperine content during maturation.

## 5 Experimental

### 5.1 Collection and Conservation of Plant Material

**FIGURE 8:**
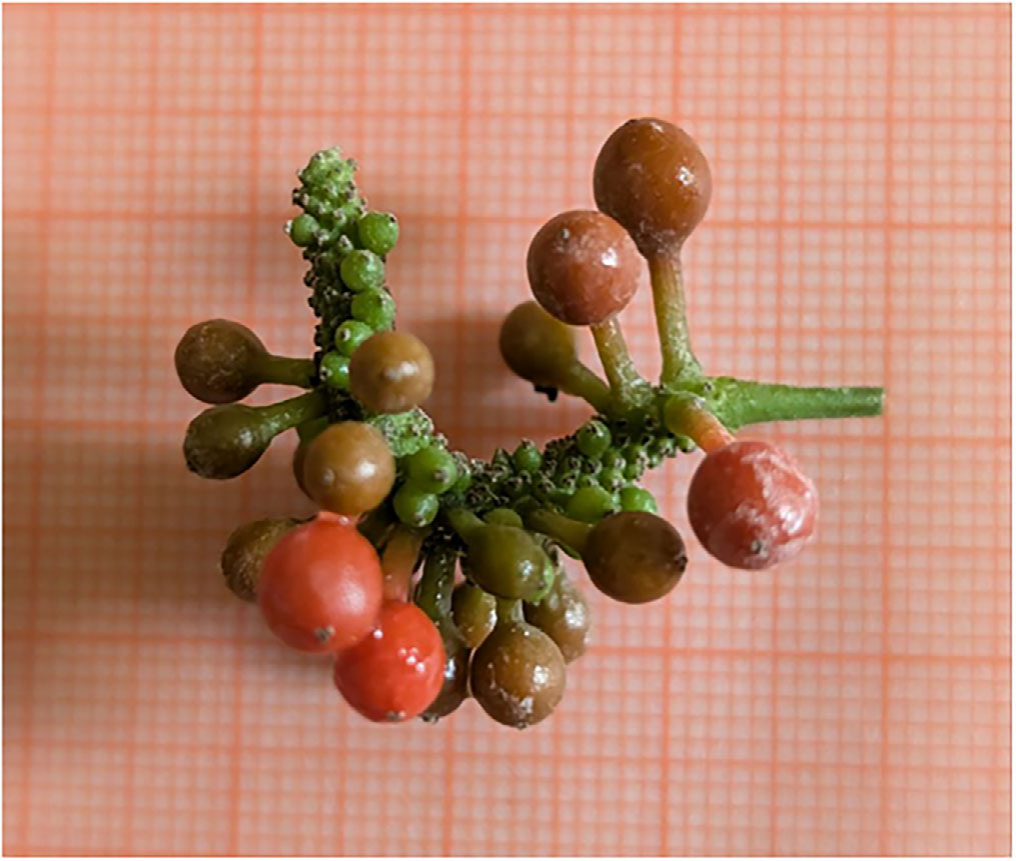
*Piper malgassicum* drupes at different phases of ripeness: green (unripe fruit), reddish (ripe fruit), and red (overripe).

The drupes of *P. malgassicum* were collected at different stages of development in the wild in Ambohimitombo, in the Ambositra district (Madagascar) from July to August 2017 (Figure 8). Drupes at different stages of ripening were collected from the aerial part of the plants, washed with tap water, and dried in the shade. The selection of the drupes was performed based on the colour of the epicarp, indicating different phases of ripeness in 50% ethanol: unripe fruit (green/greenish‐orange colour); ripe fruit (orange/reddish‐orange colour); overripe fruit (red colour).

The fresh samples, stored refrigerated at 4°C, were directly delivered from Madagascar. The plant drug was subjected to both microscopic and phytochemical analyses. Some of the ripe fruits were also dried in an oven at 35°C for further analysis.

The collection of material was very limited since *P. malgassicum* is a rare endemic of Madagascar. The sampling of the plant material respected the ethical guidelines of collection in the wild since the collection was part of a project for the reduction of the impact of the commerce of voatsiperifery pepper by directly growing *P. malgassicum* in the forest.

The plant material (conserved in the Tropical Herbarium FT, see below) recognition was carried out by comparison with the herbarium samples of *P. malgassicum* stored at the Tropical Herbarium of Florence (FT, Centro Studi Erbario Tropicale, Università degli Studi di Firenze) [4].

### 5.2 Micromorphological Analysis

#### 5.2.1 Light Microscope

Drupes of *P. malgassicum* were fixed in 50% ethanol (v/v) and at the three stages of ripeness were used for the micromorphological analysis. Ten specimens in total for each stage were used to evaluate the level of variability in the fruit morphoanatomy and the distribution and histochemistry of the secretory structures.

The samples were cut with a cryostat at ‐20°C, obtaining 30‐40 µm‐thick sections for histochemistry. The sections were stained with selective dyes for the detection of terpenoids and lipophilic substances using NADI reagent [53], for the investigation of neutral lipids using Sudan red III‐IV [54], and for identification of alkaloids using Wagner's reagent [55]. Control procedures were concurrently performed.

The sections were observed through a Leitz DM‐RB “Fluo” light/fluorescence microscope (Wetzler, Germany) equipped with a digital camera Nikon DS‐L1 camera (Tokyo, Japan).

### 5.3 Phytochemical Analysis

The drupes, collected at the three formerly described stages of ripeness and selected based on the colour of the epicarp, were separately analysed.

#### 5.3.1 Determination of the Non‐volatile Fraction

The non‐volatile fraction was extracted following the protocol described by Chandra et al. [56]. The fresh drupes were first lyophilized and then ground, while the dried samples were only ground. Then, we added 96% ethanol in a ratio of 1:10 w/v. Thus, the samples were put in an ultrasound bath for 30 min and then left for 24 h under stirring at room temperature. The extract was then filtered, and this extraction procedure was repeated two more times on the solid residue. The three extracts obtained were brought to the exact volume and adequately diluted for the quantitative analysis.

##### 5.3.1.1 HPLC/DAD analysis of alkaloids

The peak identification of piperine and its derivatives was performed by the comparison of their Rts, UV‐Vis, and MS spectra with those of commercial standards or with literature data.

The alkaloids were quantified using a four‐point calibration curve of piperine commercial standard (97% of purity from Sigma Aldrich) at λ = 330 nm, linearity range 0–0.13 µg (R^2^ = 0.99884).

HPLC analysis was carried out with an Agilent HP1100 system equipped with an autosampler, column heater module, and quaternary pump coupled to a diode array detector (DAD).

A Luna C18(2) 100A, 150 × 2.00 mm, 3 µm column (Agilent Technologies), equipped with a pre‐column of the same phase, and maintained at room temperature, was used.

The injection volume was 5 µL. The elution was performed at a flow rate of 0.2 mL/min using the following eluents: water at pH 3.2 by adding formic acid (solvent A) and acetonitrile (solvent B). All solvents used were HPLC grade.

A multistep linear solvent gradient was used starting from 0% of solvent A up to 97% and then from 97% to 70% in 5 min, up to 40% B in 10 min, up to 0% of eluent A in 8 min, with a total time of analysis of 23 min. The flow rate was 0.2 mL/min. UV–vis spectra were recorded in the range 200–600 nm, and the detector was set at 330 nm.

#### 5.3.2 Determination of the Volatile Fraction

For the extraction of the volatile fraction, we separately ground the fresh drupes at the three maturation stages with liquid nitrogen. For each stage, 0.2 g of ground material was extracted in 2 mL of n‐pentane containing tridecane (20 mg/L) as internal standard. The extraction lasted 24 h in a shaker, 5 rpm at 24°C. The extracted mixture was filtered through a syringe filter with 0.45 µm porosity.

##### 5.3.2.1 GC/MS analysis for terpene

The samples were analyzed with a GC/MS Agilent 7890 5975C MSD quadrupole mass spectrometer (Agilent Technologies, Palo Alto, CA, USA) equipped with a Gerstel MPS 2 autosampler. An Agilent DB InnoWAX (50 m; i.d., 200 µm, film thickness, 0.4 µm) column was used for analyte separation. The injected volume was 1 µL. The initial temperature was 40°C for 1 min, then increased at 5°C/min until 200°C, then 10°C/min until 260°C, and kept at 260°C for 6 min. The injector was set at 260°C, in splitless mode with He as the gas carrier at 1.2 mL/min. The spectra were acquired in the 40‐350Th range with an ionization energy of 70 eV.

The chemical compounds were identified by comparison with the NIST14/Wiley98 spectral libraries and by comparison of their Kovat retention indices with those reported in literature [57]. On the other hand, monoterpenes and β‐caryophyllene were positively identified via injection of available pure authentic standards.

The quantitative evaluation of volatile compounds concentration was performed by the direct construction of calibration lines obtained from individual pure compound solutions and a specific quantitation for the compounds positively identified. The other compounds (e.g., most of the sesquiterpenes) were quantitated using the calibration line of β‐caryophyllene constructed using the total ionic current (TIC) signal and reported as β‐caryophyllene equivalents.

The semi‐quantitative evaluation of the volatile compounds was performed by the direct comparison of peak areas of the total ionic current, as only a very approximate measurement. Since only one analysis was performed, the data should be considered only preliminary.

## Author Contributions

**Sara Falsini**: performed the histochemical staining and light microscopy observation, conceived the study, and wrote the draft of the manuscript. **Sara Ballantini**: collected and furnished the drupes needed for the work. **Alessio Papini**: performed the histochemical staining and light microscopy observation, conceived the study, designed and coordinated the experiments, wrote the draft of the manuscript, and revised the manuscript. **Gelsomina Fico**: revised the manuscript. **Claudia Giuliani**: revised the manuscript. **Enrico Palchetti**: collected and furnished the drupes needed for the work. **Massimo Gori**: collected and furnished the drupes needed for the work. **Stefano Biricolti**: carried out the phytochemical procedures and analyzed the data. **Emilio Corti**: performed the histochemical staining and light microscopy observation. **Alexander Pittella**: performed the histochemical staining and light microscopy observation, conceived the study, designed and coordinated the experiments, wrote the draft of the manuscript, and revised the manuscript. **Luca Calamai**: carried out the phytochemical procedures and analyzed the data. **Marzia Innocenti**: conceived the study, designed and coordinated the experiments, and revised the manuscript. All authors read and approved the final version of the manuscript.

## Conflicts of Interest

The authors declare no conflicts of interest.

## Data Availability

The authors have nothing to report.
